# Arbuscular Mycorrhizae Mitigate Aluminum Toxicity and Regulate Proline Metabolism in Plants Grown in Acidic Soil

**DOI:** 10.3390/jof7070531

**Published:** 2021-06-30

**Authors:** Modhi O. Alotaibi, Ahmed M. Saleh, Renato L. Sobrinho, Mohamed S. Sheteiwy, Ahmed M. El-Sawah, Afrah E. Mohammed, Hamada Abd Elgawad

**Affiliations:** 1 Department of Biology, College of Science, Princess Nourah Bint Abdulrahman University, Riyadh 84428, Saudi Arabia; mouotaebe@pnu.edu.sa; 2 Department of Botany and Microbiology, Faculty of Science, Cairo University, Giza 12613, Egypt; 3 Department of Agronomy, Federal University of Technology, Pato Branco, Paraná 85503-390, Brazil; rsobrinho@alunos.utfpr.edu.br; 4 Department of Agronomy, Faculty of Agriculture, Mansoura University, Mansoura 35516, Egypt; salahco_2010@mans.edu.eg; 5 Department of Agricultural Microbiology, Faculty of Agriculture, Mansoura University, Mansoura 35516, Egypt; ahmedelsawah89@mans.edu.eg; 6 Laboratory for Integrated Molecular Plant Physiology Research (IMPRES), Department of Biology, University of Antwerp, 2020 Antwerp, Belgium; 7 Department of Botany and Microbiology, Faculty of Science, Beni-Suef University, Beni-Suef 62521, Egypt

**Keywords:** lotus, barley, *Rhizophagus irregularis*, photosynthesis, proline metabolism, nitrogen assimilation

## Abstract

Arbuscular mycorrhizal fungi (AMF) can promote plant growth and induce stress tolerance. Proline is reported to accumulate in mycorrhizal plants under stressful conditions, such as aluminum (Al) stress. However, the detailed changes induced in proline metabolism under AMF–plant symbiosis has not been studied. Accordingly, this work aimed to study how Al-stressed grass (barley) and legume (lotus) species respond to AMF inoculation at growth and biochemical levels. The associated changes in Al uptake and accumulation, the rate of photosynthesis, and the key enzymes and metabolites involved in proline biosynthesis and degradation pathways were studied. Soil contamination with Al induced Al accumulation in tissues of both species and, consequently, reduced plant growth and the rate of photosynthesis, while more tolerance was noticed in lotus. Inoculation with AMF significantly reduced Al accumulation and mitigated the negative impacts of Al on growth and photosynthesis in both species; however, these positive effects were more pronounced in barley plants. The mitigating action of AMF was associated with upregulation of proline biosynthesis through glutamate and ornithine pathways, more in lotus than in barley, and repression of its catabolism. The increased proline level in lotus was consistent with improved N metabolism (N level and nitrate reductase). Overall, this study suggests the role of AMF in mitigating Al stress, where regulation of proline metabolism is a worthy mechanism underlying this mitigating action.

## 1. Introduction

Aluminum (Al^3+^) toxicity is a major growth-limiting factor that restrains agronomic performance in acidic soils worldwide. About 25–80% of crop productivity loss is ascribed to Al effects [1,2]. In addition to the acidity that occurs naturally in soils, the use of nitrogen fertilizers also potentiates acidification processes through the conversion of N-ammonium into nitrate by soil bacteria, releasing hydrogen (H^+^), reducing the pH of the soil, and increasing the availability of Al^3+^ for plants [3,4]. In acidic soils (pH ≤ 6), Al^3+^ inters into plant roots, where it inhibits root cell division and elongation [5,6]. Al^3+^-stressed plants also experience a great reduction in the assimilation and uptake of water and nutrients, which consequently reduces their productivity [7,8]. The accumulation of Al in leaves directly induces specific stress responses in developmental, physiological, and biochemical processes. It disrupts the cell wall and plasma membrane structure, signal transduction, and nucleotide/phosphate homeostasis and alters the antioxidant and osmo-regulation status of plants [9,10]. Despite the agricultural impact of Al in soils, knowledge of the mechanisms of plant tolerance against Al toxicity is rather fragmentary.

Differences in responses to Al toxicity may originate from species differences, as different plant groups tend to react differently to heavy metal stress at different metabolic and physiological levels. For example, legumes, such *Lotus corniculatus*, show high tolerance to aluminum stress [11]. *Lotus corniculatus* is one of the most economically and ecologically important lotus species because it can effectively grow in polluted soils and thus is used for contaminated area restoration. However, grasses, such as barley (*Hordeum vulgare* L.), one of the major world crops based on production, are sensitive to Al toxicity. In this context, Ma et al. [12] screened about 600 barley lines from various regions of the world for their responses to Al toxicity and found that most screened lines are sensitive to Al. Thus, it is important to understand how legumes and grasses differentially respond to Al accumulation in soil.

Plant tolerance to Al stress and soil acidity could be strengthened by symbiosis with arbuscular mycorrhizal fungi (AMF) [13,14,15]. AMF can provide a wide range of benefits to the host plant, such as forming a unique mycelial network, connecting plants of different species, regulating their growth, and allowing access to nutrients that would otherwise be unavailable [16]. AMF can also act as a physical barrier, capable of filtering and immobilizing toxic elements, in both extra- and intraradical fungal structures, thus mitigating the hazardous impacts of these phytotoxic elements [14,17]. From the metabolomic point of view, several mechanisms, direct and indirect, are suggested whereby AMF can stimulate plant growth and reduce the phytotoxicity of heavy metals [18,19], even in acidic soils that contain toxic levels of Al^3+^ [14,20]. For instance, AMF can improve antioxidant defenses and the production of low-molecular-weight compounds, such as proline (Pro) [7,21]. Pro is one of the compatible solutes for osmotic adjustment, acts as a molecular chaperone stabilizing proteins and membranes under stress, and acts as a scavenger for reactive oxygen species (ROS) [22]. Pro is a key amino acid involved in a wide array of plant physiological and developmental processes [23,24]. In addition to its role in protein synthesis, it is known to act in stress mitigation [23,24]. Moreover, changes in Pro metabolism may affect the cellular redox status, prompting metabolic adjustments [25]. Pro can also contribute to buffering cytosolic pH and represents a source of energy, carbon, and nitrogen for plant growth after stress relief [23,26]. Pro biosynthesis in plants is associated with both the glutamate pathway and the ornithine (Orn) pathway [27]. In the glutamate pathway, Pro is synthesized in the cytoplasm, where glutamate is converted to 1-pyrroline-5-carboxylate (P5C) by Δ 1-pyrroline-5-carboxylate synthase (P5CS), which, in turn, is converted to Pro by Δ 1-pyrroline-5-carboxylate reductase (P5CR). The pathway from Orn-to-Pro production occurs in the mitochondria, where Orn is transaminated by Orn-δ aminotransferase (OAT), forming P5C and glutamate-semialdehyde, ending the transformation into Pro [23,27]. Although several studies have pointed to the accumulation of proline in mycorrhizal plants suffering metal stress, the detailed proline metabolism under combined metal and AMF treatments has been rarely investigated [22,28]. Therefore, the current study aimed to assess the detailed changes in Pro metabolism in AMF–plants influenced by Al^+3^ toxicity by monitoring the changes in the majority of metabolites and enzymes in the pathways of Pro biosynthesis and catabolism. To highlight species-specific responses, the role of AMF–plant symbiosis in the response of two different functional group species, i.e., barley (grass) and lotus (legume), was investigated.

## 2. Materials and Methods

### 2.1. Experimental Setup, Plant Materials, and Growth Conditions

Homogeneous seeds of barley (*Hordeum vulgare L.* cv Giza 129) and lotus (*Lotus corniculatus* L cv Giza 171) were collected from the Agricultural Research Center, Giza, Egypt. Uniform and healthy seeds were sterilized by immersion for 20 min in sodium hypochlorite (35%, *v*/*v*). After that, the seeds were germinated in Petri dishes and later transplanted to pots containing artificial soil with 65% water capacity and a pH of 5, adjusted with CaCO_3_. AlCl_3_ solution was mixed with the soil in the following proportions: 0 (control) and 25 (stress) mg Al^+3^/kg soil. For AMF treatment, the AMF spores used were *Rhizophagus irregularis* MUCL 41833; these were obtained from the in vitro collection of Glomeromycota (GINCO) (www.mycorrhiza.be/ginco-bel accessed on 2 February 2021). The inoculants of AMF were prepared using the methodology of El-Sawah et al. [29]. Briefly, AMF spores were grown for 6 months on the roots of *Sorghum sudanenses* Pers., and 5 g of trapped soil per plant or per pot (approx. 50 spores per 1 g soil) was added to each pot. The inoculum was inserted before sowing, at a depth of 5 cm below the surface, thus producing mycorrhizal treatment. The pots with no mycorrhiza (control) received equal amounts of autoclaved inoculum to guarantee the supply of the same nutrients. Then, control and treated soils were incubated in a growth chamber (60/70% air humidity, 16/8 h day/night photoperiod, 21/18 °C of air temperature, and 150 µmol m^−2^ s^−1^ light intensity). The plants were then grown in four experimental settings: (1) AMF + 0 mg Al^+3^/kg soil (control); (2) soil inculcation with *Rhizophagus irregularis* MUCL 41833 (approx. 50 spores per 1 g soil) + 0 mg Al^+3^/kg soil (AMF); (3) AMF + 25 mg Al^+3^/kg soil (Al); and (4) AMF + 25 mg Al^+3^/kg soil (Al+AMF).

Each of the four groups was composed of 15 pots containing 10 plants each. The soils were watered daily, keeping the water content at 65% and the pH at 5. Plants were harvested 4 weeks after planting in pots. To determine the dry mass and analyze the elements, some organs were harvested and washed carefully with double-distilled water and gently wiped with paper towels. They were then placed to dry at 75 °C until they reached a fixed weight. The remaining samples were instantly frozen in liquid nitrogen and kept in a −80 °C freezer for future biochemical analyses. The entire experiment was repeated twice.

### 2.2. Photosynthetic Rate

The photosynthetic rate was evaluated using fully mature leaves, and the variables evaluated were light saturation, gas exchange, and the photosynthetic rate. These parameters were measured using a portable photosynthesis system (LI-COR LI-6400; LI-COR Inc., Lincoln, NE, USA) according AbdElgawad, et al. [30]. The photochemical efficiency (Fv/Fm) of non-cyclic electron transport in PSII was evaluated on 5 or 6 leaves adapted to the dark for 30 min using a fluorimeter (PAM2000; Walz, Effeltrich, Germany).

### 2.3. Mycorrhizal Parameters

Mycorrhizal colonization was verified according to the methodology of Phillips and Hayman [31]. Briefly, about 0.5 g of fresh roots was clarified with hydrogen peroxide + potassium hydroxide (10%) in a ratio of 1: 1 (*v*/*v*) and then subjected to 0.05% trypan blue dye in lactoglycerol. The roots were then evaluated under the microscope (40×) to assess the presence of colonization. The colonization rate was calculated using the gridline intersect methodology. The abundance of arbuscules in the root system was calculated using the number of root arbuscules cm-1 according to Giovannetti and Mosse [32]. The length of the hyphae was determined by the method of Andrade et al. [33].

### 2.4. Determination of Aluminum Content in Soil and Plant Tissue

The aluminum level was evaluated in the soil and in the tissues of barley shoots. Al content was determined according to Giannakoula et al. [34]. The rhizosphere was obtained by shaking the roots gently to separate them from the soil, and then the soil attached to the thin roots (0–2-mm-thick layer) was obtained gently by brushing. Then, 0.1 g of plant tissues and 5 g of soil were digested in a 4:1 (*v*/*v*) nitric acid/perchloric acid (HNO_3_/HClO_4_) solution to be ready for analysis by inductively coupled plasma–atomic emission spectrometry (ICP-AES). After extraction, the aluminum content was determined using ICP-AES (PerkinElmer Optima 3300XL; PerkinElmer, Waltham, MA, USA). The internal rhodium standard was used with the addition of samples and calibration solutions.

### 2.5. Amino Acid and Nitrogen Measurements

The extraction of amino acids was performed using sprouts homogenized in 1 mL of 80% (*v*/*v*) aqueous ethanol, supplemented with norvaline, to control the loss of amino acids during extraction. Amino acid content was assessed using the Waters Acquity UPLC-tqd system (Waters Corporation, Milford, MA, USA) along with a 2.1 × 50 amide BEH column, as described by AbdElhawad et al. [35]. Nitrogen levels were assessed using a CN element analyzer (NC-2100; Carlo Erba Instruments, Milan, Italy).

### 2.6. Enzyme Activity Assays

The activities of pyrroline-5-carboxylate synthase (P5CS), pyrroline-5-carboxylate reductase (P5CR), glutamine synthetase (GS), and Pro dehydrogenase (ProDH) were evaluated in shoot system tissues after extraction in a buffer solution containing Tris-HCl (50 mM, pH 7.4) [36,37,38]. Arginase (ARG) and Orn aminotransferase (OAT) were evaluated after their extraction in a buffer solution containing potassium phosphate (50 mM, pH 7.0). In the P5CS evaluation, the NADPH-dependent conversion of glutamate to 5-semialdehyde glutamate was evaluated. ATP was added to the mixture to ensure glutamate kinase (GK) activity. GK activity was not measured separately. ProDH and OAT activities were evaluated according to Sakuraba et al. [39] and Charest and Phan [40]. The arginase and Gly-NaOH reaction mixture was evaluated according to the methodology of Nuzum and Snodgrass [41]. NADH nitrate-dependent reductase activity (NR, EC 1.7.1.1) was evaluated with a mixture of KPO_4_ buffer solution (pH 7.6) containing 100 mM KNO_3_ and 1 mM NADH, and the oxidation of NADH in A340 was also evaluated. Nitrate reductase (NR) activity was assayed according to the method described by AbdElgawad et al. [42].

### 2.7. Statistical Analysis

All the data were subjected to one-way analysis of variance (ANOVA). Tukey’s multiple-range test (*p* < 0.05) was carried out as the post hoc test for mean separations. The number of replicates for each vegetal species was three (*n* = 5). Principal component analysis (OriginLab 9, Northampton, MA, USA) and cluster analysis (Pearson distance metric; MultiExperiment Viewer (MeV)™ 4 software, La Jolla, CA, USA) of all results were performed.

## 3. Results

### 3.1. AMF Colonization and Hyphal Growth

Mycorrhizal colonization was highly affected by different tested treatments in both plants; however, no mycorrhizal colonization was observed in the roots of plants that were not inoculated with AMF (Table 1). The roots of barley and lotus were extensively colonized by AMF. Nonetheless, in the presence of aluminum, AMF colonization significantly reduced by 26.30% and 30.59% in barley and lotus, respectively. Further, the hyphal length in the rhizosphere soil of both plants reduced significantly by 27.37% and 46.44% in barley and lotus, respectively. In addition, the number of arbuscules in roots reduced in the presence of Al in the lotus plant but not in the barley plant.

### 3.2. Plant Biomass, Photosynthesis, and Al Accumulation

The mean data regarding plant biomass, rate of photosynthesis, and Al accumulation, as affected by Al and AMF applications, are presented in Table 2. The obtained results showed that AMF treatment enhanced the growth of barley and lotus plants significantly. Under this treatment, the plants had an increase of 18% and 16% in the fresh mass and 52% and 18% in the dry mass in relation to the control treatment for barley and lotus plants, respectively (Table 2). Moreover, there was a significant change in photosynthesis after AMF treatment, with an increase of 18% for lotus plants; however, there was no significant change in photosynthesis for barley plants (Table 2). The treatment with Al increased the accumulation of Al ions in the tissues of barley and lotus plants and decreased the fresh mass, dry mass, and photosynthesis compared with individual mycorrhizal treatment (Table 2). For barley plants, Al reduced approximately 60% of fresh and dry masses, with a 70% reduction in photosynthesis compared with the control. For lotus plants, the presence of Al in the soil decreased the fresh mass, dry mass, and photosynthesis at mean values of 50% when compared with the control; this reduction was lower than that observed for barley. In addition, the treatment (Al + AMF) mitigated the negative effects of aluminum accumulation in both plants; however, this recovery was more pronounced in barley plants. Under Al + AMF treatment, the fresh mass of barley plants was 73% higher than under Al treatment. The same behavior was also observed for the dry mass, with an increase of 88%, and photosynthesis, with an increase of 100%, in relation to Al treatment. However, the increase was more than 40% for the fresh and dry masses for lotus plants (Table 2).

### 3.3. Proline Biosynthesis: Glutamine Pathway

**Figure 1:**
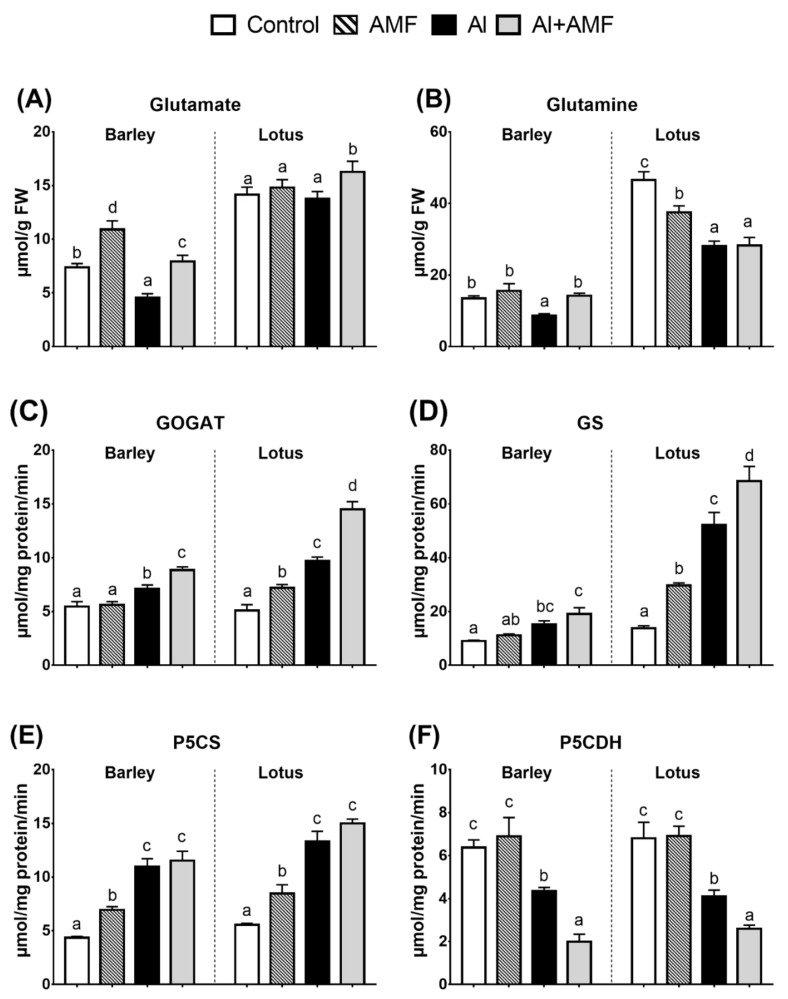
Proline biosynthesis (glutamate pathway). Changes in metabolic level and related enzyme activities: impact of arbuscular mycorrhizal fungi (AMF), Al (25 mg Al kg^−1^ soil), or their combination. Panels show concentrations of glutamate (**A**), glutamine (**B**), and the activity of glutamate synthase (GOGAT; (**C**)), glutamine synthetase (GS; (**D**)), P5C synthase (P5CS; (**E**)), and D1-pyrroline-5-carboxylate reductase (ProDH) (**F**). Different letters in each graph represent significant differences between the three treatments in each species (Tukey’s test; *p* < 0.05; *n* = 5).

The data on P5CS, P5CR, and Pro levels in barley and lotus plants are presented in Figure 1. AMF inoculation did not significantly affect proline biosynthesis via the glutamine pathway in barley, but it increased proline biosynthesis in lotus by increasing the activities of GOGAT, GS, and P5CS. Consistent with the increase in GS and GOGAT activities, glutamine content decreased under AMF treatment (Figure 1). Al treatment increased Pro biosynthesis in barley and lotus by increasing the activities of GS, GOGAT, and P5CS enzymes in both plants, with a concomitant decrease in glutamine content (Figure 1). Co-application of Al and AMF treatments strengthened the impact of Al on GS and GOGAT enzyme activities, particularly in lotus. In contrast, AMF reduced the Al impact on glutamine and glutamate concentration in barley and did not affect P5CS enzyme activity in both species. The produced glutamate 5-semialdehyde was spontaneously converted to P5C, which was catabolized to glutamate by P5CDH enzyme. The activity of P5CDH did not alter in both AMF-treated species, but it decreased under Al treatment, and the co-application of AMF and Al further strengthened this impact.

### 3.4. Proline Biosynthesis: Ornithine Pathway

**Figure 2:**
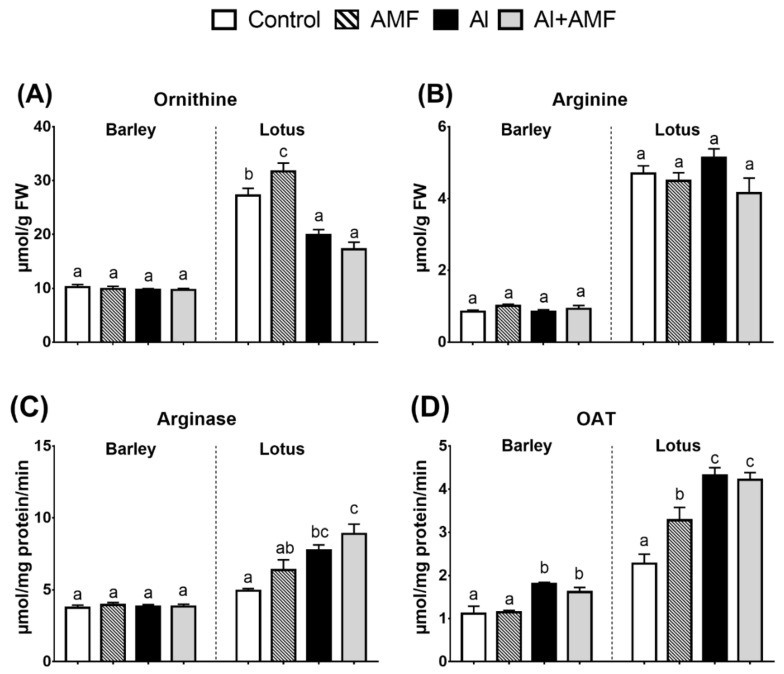
Proline biosynthesis (ornithine pathway). Changes in metabolic level and related enzyme activity in barley and lotus plants: impact of arbuscular mycorrhizal fungi (AMF), Al (25 mg Al kg^−1^ soil), or their combination. Panels show concentrations of ornithine (**A**), arginine (**B**), and the activity of arginase (**C**) and ornithine aminotransferase (OAT; (**D**)). Different letters in each graph represent significant differences between the three treatments in each species (Tukey’s test; *p* < 0.05; *n* = 5).

Arginine (Arg) and orninthine (Orn) provide precursors for the Pro synthesis pathway (Figure 2). AMF inoculation had no significant effects on the level of Arg in both species, whereas arginase and orninthine aminotransferase (OAT) significantly increased in lotus (Figure 2). Al stress had no impact on the arginine content in both treated species; however, Orn content reduced in lotus but not in barley. Co-application of AMF and Al did not significantly change the Al effect on the metabolic level of Arg and Orn. OAT activity increased in AMF- and Al-treated barley plants and to a greater extent in treated lotus plants (Figure 2), but this increase was not observed for arginase activity.

### 3.5. P5C–Pro Metabolism Cycle

**Figure 3:**
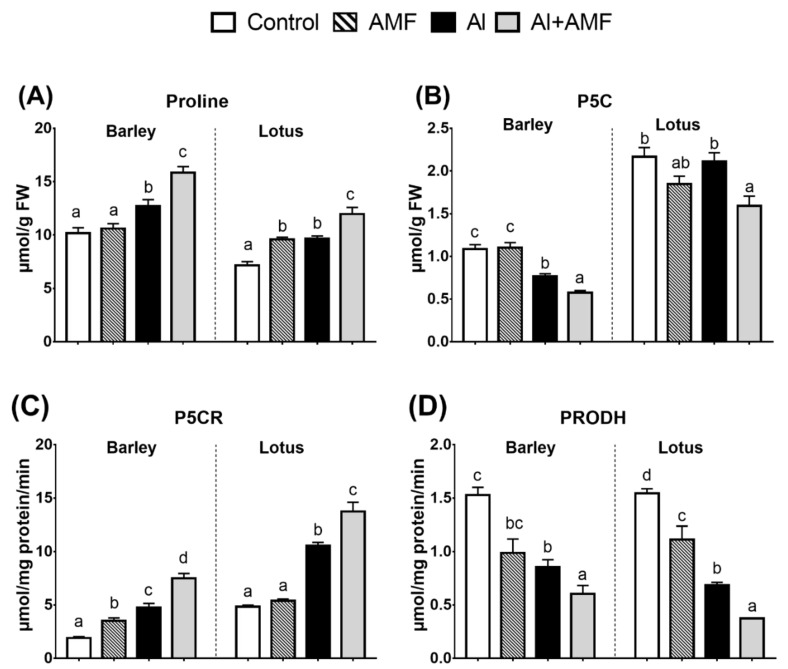
Pro biosynthesis (P5C–Pro metabolism cycle). Changes in metabolic level and related enzyme activities: impact of arbuscular mycorrhizal fungi (AMF), Al (25 mg Al kg^−1^ soil), or their combination. Panels show concentrations of proline (**A**) and its immediate precursor P5C (**B**) and the activity of pyrroline-5-carboxylate reductase (P5CR) (**C**) and proline dehydrogenase (ProDH) (**D**). Different letters in each graph represent significant differences between the three treatments in each species (Tukey’s test; *p* < 0.05; *n* = 5).

The obtained results, as shown in Figure 3, revealed that under AMF treatment, there was a significant increase in Pro only in lotus, with a slight decrease in the immediate precursor (P5C). Although there was no increase in the Pro level in barley, an increase in P5CR was recorded. In contrast, there was a significant decrease in the activity of the catabolic enzyme (ProDH) in lotus, but this decrease was little in barley (Figure 3). Al stress in general increased Pro in both species, with a decrease or no change in the P5C level in barley and lotus, respectively. In line with the high proline level in treated species, an increase in P5CR activity and a decrease in ProDH activity were observed. In all cases, AMF treatment increased the Al-stress-induced Pro increase (Figure 3). This was consistent with the observed increase and decrease in P5CR and ProDH activities in both species, respectively.

### 3.6. Nitrogen (N) Assimilation

The obtained results (Table 3) revealed that AMF inoculation had no significant impact on the nitrogen content or nitrate reductase activity in both barley and lotus plants. On the contrary, under Al treatment, nitrogen content and nitrate reductase activity decreased in both plants. Interestingly, the co-inoculation of AMF with Al recovered the negative impact of Al toxicity on the nitrogen content in both plants and on nitrate reductase in lotus only.

### 3.7. Hierarchical Clustering of Proline-Metabolism-Related Parameters

**Figure 4:**
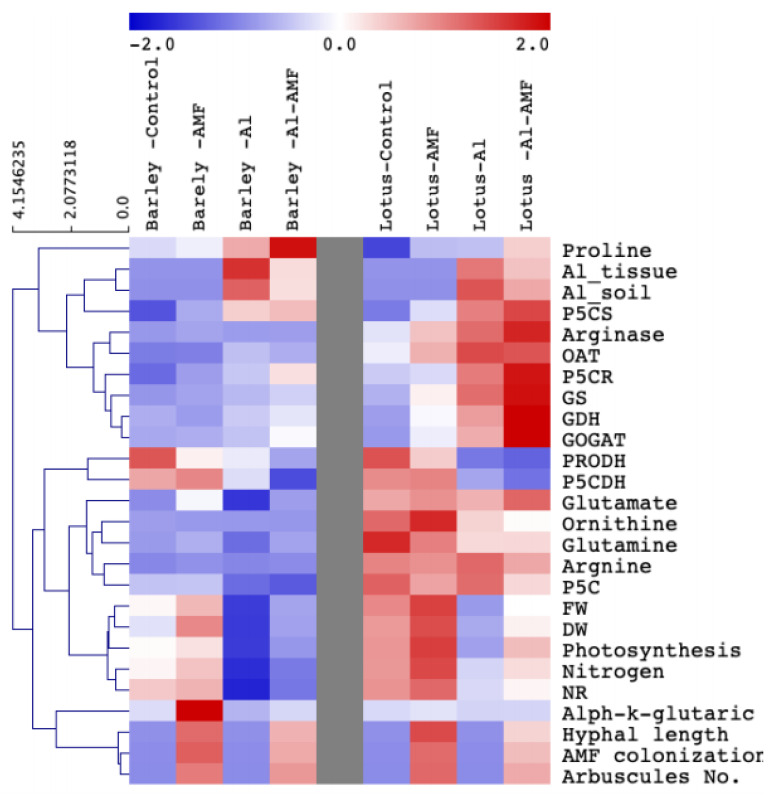
Hierarchical clustering of proline-metabolism-related parameters. The levels patterns are relatively demonstrated on the heatmap based on the mean value (*n* = 5) for each parameter. Red and blue color gradients indicate higher and lower levels, respectively. Full names are indicted in the legends of Figure 1, Figure 2 and Figure 3 and Table 1, Table 2 and Table 3.

Hierarchical clustering analysis showed divergences in proline metabolic relative values in AMF-treated barley and lotus plants under control and Al stress conditions (Figure 4). There were two major groups, with the first containing proline and its biosynthetic enzymes (GS, GDH, GOGAT, P5CR, arginase, and OAT) that were higher in Al-stressed plants, particularly lotus plants. In most cases, the increase in Pro biosynthetic enzymes was significantly induced by AMF and Al co-application, particularly in lotus. However, the second group contained plant growth- and physiology-related parameters, AMF colonization, arbuscule number and Pro catabolic enzymes (ProDH and P5CDH), proline percussors (e.g., glutamine, glutamate, ornithine, and arginine), and nitrogen assimilation. The parameters in group 2 were in general higher in non-Al stressed plants, particularly in lotus. Moreover, AMF inculcation significantly reduced the negative impact of Al stress in both species.

## 4. Discussion

### 4.1. Al Greatly Inhibits Growth and Photosynthesis in Both Plants, with Lotus Bring More Tolerant

The accumulation of Al in plants is known to induce a hazardous impact at developmental, physiological, and biochemical levels. For instance, the negative effects of Al on plant biomass have been previously reported [5,6,43]. Al accumulated in the two species but to a greater extent in barley, resulting in impairment in root growth (data not shown). In contrast, lotus was less sensitive to the impact of Al stress compared with barley. These results suggest that uptake of Al by roots and/or its penetration into the stele is lower in lotus, resulting in a lower content in leaves, which can be considered a kind of adaptation mechanism whereby plants protect the photosynthetic organs from Al toxicity [44,45,46]. In this regard, a previous study reported that cereal plants accumulate high amounts of Al, particularly in their roots [47]. The higher accumulation of Al in plant roots can induce several morphological and biochemical changes, such as deformation of root hairs and the root apex, causing inhibition of roots to uptake essential nutrients, consequently affecting plant growth [7,8]. To further understand the inhibition caused by Al stress on plant growth, the photosynthetic rate was measured in both treated plants (Table 2). It is well known that the photosynthesis process is extremely sensitive to heavy metal exposure [48]. As such, Al interferes with light absorption, electron transport, gas exchange, pigments, carbohydrates, photo-protective systems, photosynthetic enzymes, and CO_2_ assimilation [49,50,51]. A previous study demonstrated that AMF improve the growth and photosynthesis rate of C4 (*Hemarthria altissima*) and C3 (*Leymus chinensis*) species via promoting their ROS-scavenging systems [52,53] or by accumulating greater osmoprotectants, such as Pro [54]. In the present study, lotus and barley plants differentially responded to Al stress, where the photosynthetic rate of barley was more affected compared with that of lotus. Accordingly, the fresh and dry masses of barley plants decreased under Al stress by 60% and 71%, respectively, compared with the control plants. However, for lotus plants, the average values of dry mass and rate of photosynthesis reduced by only 46% and 53%, respectively. Interestingly, AMF diminished the inhibition role of Al stress in both plant species (Table 2); therefore, biomass production increased under the effect of AMF–plant symbiosis, which might be due to the potentiality of AMF to increase nutrient uptake [53]. Moreover, the use of photosynthates could contribute to increasing the hyphal growth of AMF, which ensure symbiotic Pi acquisition by the host plant under Al stress, which, in turn, helps in maintenance of photosynthesis and C flow to the root system [20].

### 4.2. AMF Decreased Al Accumulation and Alleviated its Negative Impact on Growth and Photosynthesis

It is well known that AMF have a symbiosis relationship with plants, in which the host provides AMF with energy through carbon assimilation; therefore, environmental factors, including heavy metal stress, such as Al stress, can affect AMF by affecting the activities of their host [55]. The interaction between AMF and plants can enrich soil resources with essential nutrients that help plants to effectively respond to different environmental factors [56]. Several studies have reported the effects of environmental factors on the effectiveness of AMF in improvement of plant growth [29]; since AMF are attached to plant roots, they receive a lower amount of photosynthates under Al stress. The present study reported that mycorrhizal growth markedly decreased in lotus compared with barley under Al stress (Table 1), demonstrating that the impact of Al levels on mycorrhizal growth seems to be dependent on the plant species.

A few studies have reported that AMF colonization ameliorates Al toxicity, leading to improved plant growth and stress tolerance [57,58]. In addition to the colonization, the interaction of AMF with host roots may extend the efficacy of Al resistance by upregulating the resistance mechanisms of host plants, such as enhancing antioxidant defense systems and supporting osmotic adjustment [7,21]. Further, AMF could provide new mechanisms for Al exclusion and detoxification, such as metal immobilization in extra- and intraradical fungal structures [17,20]. Further, AMF–plant symbiosis can facilitate nutrient acquisition by altering the availability and/or speciation of Al in the rhizosphere [55]. In the present study, AMF treatment markedly reverted the effects of Al stress on plant growth, resulting in a significant increase in fresh and dry masses, especially in barley (Table 2). This could be related to the decrease in Al accumulation in plant tissues, which might be due to the ability of AMF to reduce the absorption and translocation of toxic metals by the host plants [59]. In this context, Wu et al. [17] reported the immobilization of chromium on the fungal surface and in the arbuscules and intraradical mycelium within mycorrhizal roots.

### 4.3. Proline Metabolism in Lotus Is More Responsive to Al Than in Barley

Proline, a multifunctional stress-induced metabolite, is reported to accumulate in mycorrhizal plants exposed to various types of abiotic stresses [7,21]. In the current study, to reveal the mechanism behind AMF-induced proline accumulation, detailed changes in proline biosynthesis and degradation pathways were investigated in host plants under Al toxicity. As a first species-specific response to Al stress, although the Glu pathway for Pro biosynthesis was promoted in both species, the enhancement was more pronounced in lotus (Figure 1 and Figure 4). In the present study, AMF significantly improved P5CS activity under Al stress. This enzyme is commonly considered a key regulator of the Glu pathway [30,60]. However, our result is inconsistent with a previous study reporting that AMF seedlings show significantly lower P5CR and P5CS activities than non-inoculated seedlings [61], indicating that the decrease in Pro accumulation in AMF seedlings is closely related with the decrease in glutamate synthetic pathways and the increase in Pro catabolism. In the current study, OAT activity considerably increased only in lotus plants, suggesting a species-specific contribution of Pro through the Orn pathway under Al stress. At the degradation level, ProDH re-oxidizes Pro to P5C, so lower activity contributes to Pro accumulation. Inhibition of ProDH was also previously observed in other species under different stress conditions [62]. Herein, there was a pronounced increase in Pro in lotus plants, which might be due to the extra Pro supply through the Orn pathway, and a stronger inhibition of Pro degradation. The participation of each pathway in the stress-induced Pro accumulation is plant species and stressor dependent [63,64]. For instance, Pro accumulation in rice stressed by Cu was induced through the Orn pathway [65]. However, in tobacco grown under Cu and drought stress, both the Glu and Orn pathways for Pro biosynthesis were upregulated [28]. Moreover, other studies have indicated the differential accumulation of Pro in soybean and rice genotypes as being responsible for the genotype-specific tolerance to heavy metal toxicity [65,66]. Notably, genotype sensitivity differences to other abiotic stressors in plants have also sometimes been linked to differences in Pro levels [59,67].

Since Pro metabolism is closely connected to nitrogen metabolism [68], the present study suggested that increased nitrogen assimilation in lotus improved the ability to further elevate Pro levels to increase tolerance to Al stress, consistent with previous studies reporting that the external addition of nitrogen results in elevated Pro [69,70]. In several cases, the increase in nitrogen might be due to the Orn pathway [69], supporting our speculation. AbdElgawad et al. [30] demonstrated that Pro accumulation in grasses and legumes under drought stress occurs mainly via the Glu pathway in grasses and the Orn pathway in legumes. They attributed this difference to the better nitrogen status in legumes than in grasses. To further validate our hypothesis, we measured the N content and nitrate reductase activity in both barley and lotus plants. We found higher N content and NR activity in lotus, especially under stress conditions. NR activity, N levels, and N/C ratios were less reduced in the tolerant rye line, which showed an upregulated Orn pathway [71]. Overall, Pro content strongly increased in barley and lotus, although through different mechanisms, and possibly is directly linked with the N status in plants.

AMF further enhanced Pro accumulation in Al-treated plants through upregulation of the glutamate pathway and downregulation of Pro degradation. Proline, the key osmolyte contributing toward osmotic adjustment, was comparatively higher in AMF-treated plants enduring Al stress. The higher Pro levels in AMF-treated plants were due to upregulation of the glutamate pathway and downregulation of Pro degradation. Further, such enhanced proline accumulation was consistent with the improved N content in mycorrhizal plants grown under Al stress (Table 3). These results reveal that mycorrhizal-treated plants acquire higher osmotic adjustment to support tolerance to Al stress, which is likely related to Pro. Similarly, a higher Pro accumulation in AMF plants under drought was observed in *Lactuca sativa* [72], *Oryza sativa* [73], and *Macadamia tetraphylla* [74]. AMF colonization is widely believed to stimulate mineral nutrient uptake in plants, e.g., N, P, and K [16]. In this regard, AMF are effectively used to enhance the biological nitrogen fixation and phosphate solubilization processes in soil and the rhizosphere [75]. In addition, mycorrhizal inoculation significantly enhanced dehydrogenase activity, which could be attributed to the ability of AMF to improve the physical and chemical properties of soil, especially the soil structure, enhancing the organic matter decomposition and remobilizing nutrients, including nitrogen, in the rhizosphere and soil [29]. Due to the close connection between Pro and N metabolism [68], enhanced N uptake could enhance Pro accumulation Pro [69,70].

## 5. Conclusions

The presence of Al in the soil increases its uptake, which consequently impairs barley and lotus growth and photosynthesis; however, lotus is more tolerant. To reduce Al toxicity, both plants species accumulate high levels of Pro, with some species-specific responses in Pro biosynthesis pathways. Inoculation with AMF reduces the levels of aluminum accumulation in plant tissues and consequently recovers the negative impact of Al on growth and the rate of photosynthesis, especially in barley. Further, AMF treatment promotes the accumulation of Pro and its percussors through activating Pro biosynthesis and reducing its catabolism. The use of AMF for inoculation of barley and lotus plants demonstrates great beneficial potential for protecting plants against Al stress. This knowledge can help plant future crops in soils contaminated by heavy metals, which today are considered useless for agriculture.

## Figures and Tables

**Table 1:** Mycorrhizal colonization and growth parameters in roots of barley and lotus under normal conditions (control) and Al stress. Values are the mean ± standard error of five independent replicates. Different letters indicate significant changes (*p* < 0.05) between AMF alone and combined AMF + Al treatment.

Parameter	Barley				Lotus			
	Control	AMF	Al	Al + AMF	Control	AMF	Al	Al + AMF
AM colonization (% per root)	0	58.35 ± 1a	0	43.0 ± 1.45b	0	55.9 ± 1.6a	0	38.8 ± 1.7b
Hyphal length (mm/g soil)	0	1943 ± 98a	0	1411.2 ± 12b	0	2168 ± 198a	0	1161 ± 101b
Arbuscules (number/cm root)	0	4.8 ± 0.703a	0	4.4 ± 0.34a	0	5.1 ± 0.7a	0	4.1 ± 0.3b

**Table 2:** Impact of arbuscular mycorrhizal fungi (AMF), Al (12 mg Al kg^−1^), or their combination on fresh and dry masses (g plant^−1^) and rate of photosynthesis (µmol CO_2_ m^−2^ s^−1^) in barley and lotus and on the accumulation of Al^+3^ in soil (mg g^−1^ soil) and in tissues of plant shoot (mg g^−1^ DW). Values are presented as the mean ± standard error of at least five independent replicates. Different letters within the same plant indicate significant differences between means (Tukey’s test; *p* < 0.05).

Parameter	Barley				Lotus			
	Control	AMF	Al	Al + AMF	Control	AMF	Al	Al + AMF
Fresh weight	2.03 ± 0.1c	2.4 ± 0.15d	0.82 ± 0.03a	1.43 ± 0.14b	2.68 ± 0.05c	3.11 ± 0.2d	1.38 ± 0.12a	1.97 ± 0.21b
Dry weight	0.44 ± 0.02b	0.67 ± 0.06c	0.18 ± 0.02a	0.34 ± 0.07b	0.64 ± 0.01c	0.76 ± 0.08d	0.35 ± 0.01a	0.51 ± 0.08b
Rate of photosynthesis	0.14 ± 0.01c	0.15 ± 0.01c	0.04 ± 0.02a	0.08 ± 0.01b	0.19 ± 0.01b	0.23 ± 0.01bc	0.09 ± 0.01a	0.17 ± 0.02b
Al in tissues	ND	ND	164.84 ± 22.95b	76.48 ± 2.53a	ND	ND	128.62 ± 4.32b	90.11 ± 11.41a
Al in soil	ND	ND	15.51 ± 0.38b	08.86 ± 1.19a	ND	ND	16.24 ± 1.62b	11.72 ± 1.02a

ND: non-detected.

**Table 3:** Nitrogen content and nitrate reductase (NR) in barley and lotus shoot under normal (control) and Al stress conditions. Values are the mean ± standard error of five independent replicates. Different letters indicate significant changes (*p* < 0.05) between AMF alone and combined AMF + Al treatment.

Parameter	Barley				Lotus			
	Control	AMF	Al	Al + AMF	Control	AMF	Al	Al + AMF
Nitrogen	218.3 ± 12c	238 ± 17c	133 ± 15a	163 ± 21b	255 ± 19c	285 ± 26c	198 ± 16a	228 ± 13b
Nitrate reductase	443 ± 28b	453 ± 98b	311 ± 32a	351 ± 12a	468 ± 31c	488 ± 41c	378 ± 18a	421 ± 27b

## Data Availability

Data presented in this study are available on reasonable request.
